# Race/Ethnicity-Specific Association of Vitamin D and Global DNA Methylation: Cross-Sectional and Interventional Findings

**DOI:** 10.1371/journal.pone.0152849

**Published:** 2016-04-06

**Authors:** Haidong Zhu, Jigar Bhagatwala, Ying Huang, Norman K. Pollock, Samip Parikh, Anas Raed, Bernard Gutin, Gregory A. Harshfield, Yanbin Dong

**Affiliations:** 1 Georgia Prevention Institute, Department of Pediatrics, Medical College of Georgia, Georgia Regents University, Augusta, Georgia, United States of America; 2 Internal Medicine, Department of Medicine, Medical College of Georgia, Georgia Regents University, Augusta, Georgia, United States of America; Queen's University Belfast, UNITED KINGDOM

## Abstract

**Objectives:**

Understanding of the influence of vitamin D deficiency on epigenome will provide novel insights into the chronic disease risk. We tested our hypotheses that 1) vitamin D deficiency is associated with global hypomethylation and this association may be race/ethnicity dependent; and 2) vitamin D supplementation will increase global DNA methylation level.

**Methods:**

A two-stage design, cross-sectional observation followed by a 16 week randomized, double- blinded, placebo-controlled trial (RCT) of vitamin D3 supplementation, was undertaken. Global DNA methylation level (percentage of 5-methylcytosine, %5-mC) was quantified using leukocyte DNA with the MethylFlash^TM^ Methylated DNA Quantification kit (Epigentek). Global methylation data was obtained from 454 Caucasians and African Americans (42%) in the observation cohort and 58 African Americans with vitamin D deficiency in the dose responsive RCT.

**Results:**

In the cross-sectional study, African Americans had lower %5-mC than Caucasians (*P* = 0.04). A significant interaction was detected between plasma 25(OH)D and race on %5-mC (*P* = 0.05), as a positive association was observed between plasma 25(OH)D and %5-mC in African Americans (β = 0.20, *p*<0.01), but not in Caucasians (β = 0.03, *p* = 0.62). In the 16-week RCT, a dose-response benefit of vitamin D_3_ supplementation was observed for %5-mC, as indicated by a significant linear upward trend (-0.01 ± 0.01%, placebo; 0.11 ± 0.01%, ~600 IU/day; 0.30 ± 0.01%, ~2,000 IU/day; and 0.65 ± 0.01%, ~4,000 IU/day group; *P*-trend = 0.04).

**Conclusions:**

Vitamin D deficiency is associated with global hypomethylation in African Americans. Vitamin D3 supplementation increases global DNA methylation in a dose-response manner in African Americans with vitamin D deficiency.

## Introduction

Increasing evidence suggest a strong reciprocity between the vitamin D system and epigenetic mechanisms.[1] On the one hand, epigenetic modifications play a key role in the maintenance of vitamin D receptor-directed gene expression and the distortion of these mechanisms can lead to pathological conditions.[2,3] On the other hand, vitamin D regulates epigenetic events.[1]

Global DNA hypomethylation is a common epigenetic event in cancer that mostly results from hypomethylation of repetitive DNA elements. Loss of global methylation may lead to chromosomal instability, loss of imprinting, and activation of transposable elements, which lead to disturbances in the genome.[1] The impact of nutrition on epigenome has been increasingly recognized as several nutrients are well known for their impact on DNA methylation, such as folic acid, vitamin B, green tea and alcohol.[4] However, the effect of vitamin D on global methylation remains largely unknown. To date, a few studies have looked into the relationship between vitamin D status and global DNA methylation mostly in Caucasian populations and reported mix results.[5–8] However, the effect of vitamin D on global methylation in African Americans remains unexplored.

Several studies reported a race/ethnic influence on global DNA methylation.[9–11] However, whether vitamin D plays a role in global DNA methylation and whether race/ethnicity plays a role in the relationship between vitamin D status and global methylation remain unclear. Therefore, we first tested our hypothesis that low vitamin D status would be associated with global hypomethylation and this association would be race/ethnicity dependent. To further establish the causal relationship between vitamin D level and global DNA methylation in African Americans, we took advantage of a previously completed randomized placebo controlled trial (RCT) of vitamin D supplementation. We hypothesized that vitamin D supplementation would increase level of global DNA methylation in individuals with suboptimal vitamin D status.

## Methods

### Participants

#### Cross-sectional study

We first examined the relationship between vitamin D status and global methylation in a cross-sectional cohort of apparently healthy Caucasians and African Americans (n = 454) aged 14–18 years who previously participated in the Lifestyle, Adiposity and Cardiovascular Health in Youth (LACHY) study. Participants were recruited from high schools in the Augusta, Georgia area.[12]

#### Randomized clinical trials

To establish the causal effect of vitamin D on global DNA methylation in African Americans, we took advantage of our previously conducted 16-week dose responsive randomized, double-blinded, placebo-controlled trial (RCT) of vitamin D3 supplementation (NCT01583621).[13] A total of 70 young African Americans with vitamin D deficiency (defined as 25(OH)D concentrations ≤20 ng/mL) were randomly assigned to receive a supervised monthly oral vitamin D_3_ dose of 18,000 IU (~600 IU/day, n = 17), 60,000 IU (~2,000 IU/day, n = 18), 120,000 IU (~4,000 IU/day, n = 18), or placebo (n = 17). Participants were recruited from local community around the Augusta, Georgia area. Inclusion criteria were self-reported African American race, age between 13–45 years, overweight/obese, no pregnancy, no lactation, no known acute or chronic illnesses, no use of any prescription medications, birth control pills, herbal, multi-vitamin or mineral supplementations, and vitamin D deficiency at screening visit.[13] Participants in both studies were healthy, free of any acute or chronic illness. Race was determined by self-report or by a parent or guardian if subject was under 18 years of age.

### Ethics Statement

The project was approved by the Institutional Review Board at the Medical College of Georgia, Georgia Regents University (Augusta, Georgia, United States, protocol # 622505). Written informed consent was obtained from all adult subjects and from parents or guardians of those who were less than 18 years of age. Each participant was assigned a unique subject number. All data were anonymized and de-identified prior to analyses.

### Anthropometric measurements

Height and weight were obtained according to standard procedures, using a wall-mounted stadiometer (Tanita Corporation of American, Arlington Heights, IL) and calibrated electronic scale (model CN2OL; Cardinal Detecto, Webb City, MO). Prior to testing each week, the electronic scale was checked for accuracy using known weights. BMI was calculated as weight (kg) divided by height (m^2^). For subjects <18 years, the exact percentile of BMI was computed.

### Complete blood count with differential test (CBC test)

Peripheral blood was collected and brought to the clinical pathology core lab at the Medical College of Georgia within 2 hours for the complete blood count with differential, which included the total leukocyte count and percentages of peripheral blood cell types including neutrophils, lymphocytes, monocytes, eosinophils and basophils.

### 25-hydroxyvitamin D measurement

Liquid chromatography tandem mass spectroscopy (LC-MS/MS) was used to measure fasting plasma levels of 25(OH)D_2_ and 25(OH)D_3_ in the LACHY study as described previously.[14] The detection limits for 25(OH)D_2_ and 25(OH)D_3_ were 10 nmol/L. The intra-assay coefficient of variation for 25(OH)D_2_ and 25(OH)D_3_ was 6–9% and 7–11%, respectively, whereas the inter-assay coefficient of variation was 9–12% and 8–13% for 25(OH)D_2_ and 25(OH)D_3_, respectively. Regarding the assay specificity, LC–MS/MS has the advantage of being able to measure both 25(OH)D_2_ and 25(OH)D_3_ independently[15]. The levels of total 25(OH)D including 25(OH)D_2_ and 25(OH)D_3_ were used in the analysis.

Enzyme immunoassay (Immunodiagnostic Systems, Fountain Hills, AZ) was used to measure fasting serum 25(OH)D concentrations in the dose responsive RCT of vitamin D_3_ supplementation trial (NCT01583621). The intra- and inter-assay coefficients of variation (CV) were 5.6 and 6.6%, respectively. Our laboratory is certified by the vitamin D external quality assessment scheme (DEQAS), an international program monitoring accuracy of 25(OH)D measurements and by the National Institute of Standards and Technology (NIST) and NIH Vitamin D Metabolites Quality Assurance Program (VitDQAP).

### Global DNA methylation assessment

Genomic DNA was extracted from stored buffy coat samples from the two studies using Qiagen® QiAamp® DNA Mini Kit (Qiagen Inc. Valencia, CA). DNA samples with OD 260/280 and 260/230 greater than 1.8 were used for global DNA methylation measurement. Global DNA methylation level was quantified in 100 ng genomic DNA using leukocyte DNA with the MethylFlash^TM^ Methylated DNA Quantification kit (Epigentek, Farmingdale, NY) following manufacturer’s instruction. Briefly, the methylated DNA was detected using capture and detection antibodies to 5-methylcytosine (5-mC) and then quantified colorimetrically by reading absorbance at 450 nm using Bio-Tek PowerWave HT Microplate Spectrophotometer (biotek, Winooski, VT). The amount of methylated DNA is proportional to the OD intensity measured. Relative quantification was used to calculate percentage of 5-mC (%5-mC) in total leukocyte DNA following the manufacturer’s instructions. Each sample was run in duplicate. In the vitamin D supplementation trial, the pre- and posttest samples from the same participant were run on the same plate. Percentage of 5-mC data was obtained from 454 subjects in the LACHY study and from 58 subjects at both baseline and 16-week post intervention visits in the RCT of vitamin D3 (Table C in S1 File).

### Statistical analysis

For the LACHY study, normal distribution and homogeneity of variances were confirmed by Shapiro-Wilks *W* and Levene’s tests, respectively. Value of global %5-mC was log-transformed. Independent *t*-tests were conducted with these general characteristics to examine the potential differences between Caucasians and African Americans. Group differences in categorical variables were tested by using chi-square tests. Descriptive statistics for raw variables are presented as mean ± SD. Multivariate linear regression analyses were conducted to examine associations of 25(OH)D with global %5-mC. Potential confounding factors including age, sex, race, BMI and batch effect. The interactions of 25(OH)D with race was tested in the linear regression analysis.

Repeated-measures mixed-models were used with maximum likelihood estimation in an intention-to-treat analysis of each outcome measure using all available data. Base model for outcome measure (global %5-mC) included the fixed effects of intervention group (placebo, 600 IU, 2000 IU, 4000 IU) and measurement time (baseline, 16 weeks) and their interaction. The base models also controlled for baseline value. Participant nested within group was considered a random effect. The modeled covariance structure between measurement periods was unstructured, which used all available measurements on the same subject. Because significant group differences in age, gender and BMI were not detected, they were not included as covariates. *A priori* linear contrasts across the four groups of the change over time tested the dose-response effects of vitamin D supplementation. In addition, we conducted Pearson’s bivariate correlation to examine the association between change in 25(OH)D and change in global %5-mC levels or change in leukocyte composition. All statistical analyses were performed using SPSS software (Version 23, IBM SPSS Statistics, Armonk, NY), and statistical significance was set at *P* < 0.05.

## Results

### Vitamin D deficiency is associated with global hypomethylation in African Americans: cross-sectional findings

To test our hypothesis that vitamin D deficiency is associated with global hypomethylation and this association is race/ethnicity dependent, we first investigated the relationship between 25(OH)D and global methylation in a cross-sectional cohort of the LACHY study. A total of 454 Caucasian and African American subjects had both plasma 25(OH)D and global %5-mC measured. Participant characteristics are presented in Table 1. Compared to Caucasians, African Americans had higher body weight and BMI, but lower circulating 25(OH)D and global %5-mC. Vitamin D deficiency was more prevalent in African Americans than in Caucasians (Table 1).

**Table 1:** General characteristics of LACHY study participants.

	Caucasians (N = 263)	African-Americans (N = 191)	*P* value
Age	16.1 ± 0.1	16.1 ±0.1	0.79
Male/Females	130/133	84/107	0.25
Height, cm	168.7 ± 0.5	167.5 ± 0.7	0.13
Weight, kg	63.6 ± 0.9	66.9 ± 1.1	0.02
BMI, kg/m^2^	22.2 ± 0.3	23.9 ± 0.4	<0.01
Tanner stage, (1–5)	4.3 ± 0.0	4.4 ± 0.1	0.11
25(OH)D, ng/ml	38.0 ± 0.9	18.3 ± 0.6	<0.01
Vitamin D deficiency, %	2.7	66.0	<0.01
5-mC (%)	0.44 ± 0.1	0.40 ± 0.2	0.04

Data explained as mean ± SE. p value obtained by independent two-tailed *t*-test.

BMI: body mass index; 25(OH)D: 25-hydroxyvitamin D; %5-mC: percentage of global 5-methylcytosine; Vitamin D deficiency: 25(OH)D ≤ 20 ng/mL

In the entire cohort, there was a positive correlation between 25(OH)D and global %5-mC, (r = 0.09, *p* = 0.05). It remained significant after adjusting for age, sex, BMI and batch effect (r = 0.11, *p* = 0.04). Moreover, there was a significant interaction between 25(OH)D and race on global %5-mC (β = 0.27, *p* = 0.05). Stratification of the sample by race showed that the association between 25(OH)D and global %5-mC was found only in African Americans (β = 0.20, *p*<0.01), not in Caucasians (β = 0.03, *p* = 0.62) after adjusting for age, sex, BMI and batch effect.

### Vitamin D supplementation increases level of global DNA methylation: interventional findings

To further establish the causal effect of vitamin D on global methylation in African Americans, we took advantage of our previously conducted RCT of vitamin D3 supplementation (NCT01583621). Of the 70 participants who enrolled in the study, global %5-mC data were obtained from 58 participants. Table 2 presents the characteristics of the study participants of the vitamin D intervention study.

**Table 2:** Baseline Characteristics of study participants in vitamin D3 supplementation trial.

Variable	Study groups				p-value
	Placebo (n = 15)	600 IU/day (n = 12)	2,000 IU/day (n = 16)	4,000 IU/day (n = 15)	
Age (years)	28.2 ± 2.7	25.6 ± 2.9	24.7 ± 2.2	25.2 ± 2.3	0.74
Male/Female*	4/11	2/10	3/13	2/13	0.81
Height (cm)	164.6 ± 2.3	164.9 ± 2.5	163.9 ± 2.5	164.2 ± 1.9	0.99
Weight (kg)	99.4 ± 7.0	94.2 ± 4.5	99.9 ± 5.2	94.5 ± 5.2	0.83
BMI (kg/m^2^)	36.4 ± 2.1	34.7 ± 1.8	37.4 ± 1.9	35.0 ± 1.9	0.77
Serum 25(OH)D (ng/mL)	15.8 ± 1.6	13.4 ± 0.8	15.9 ± 1.1	12.9 ± 1.0	0.15
5-mC (%)	0.53 ± 0.2	0.81 ± 0.2	0.36 ± 0.1	0.41 ± 0.1	0.19

Values are presented as mean ± SE; BMI: Body mass index; 25(OH)D: Serum 25-hydroxyvitamin D; %5-mC: percentage of global 5-methylcytosin.

*: Group differences analyzed using chi-square test.

**Fig 1:**
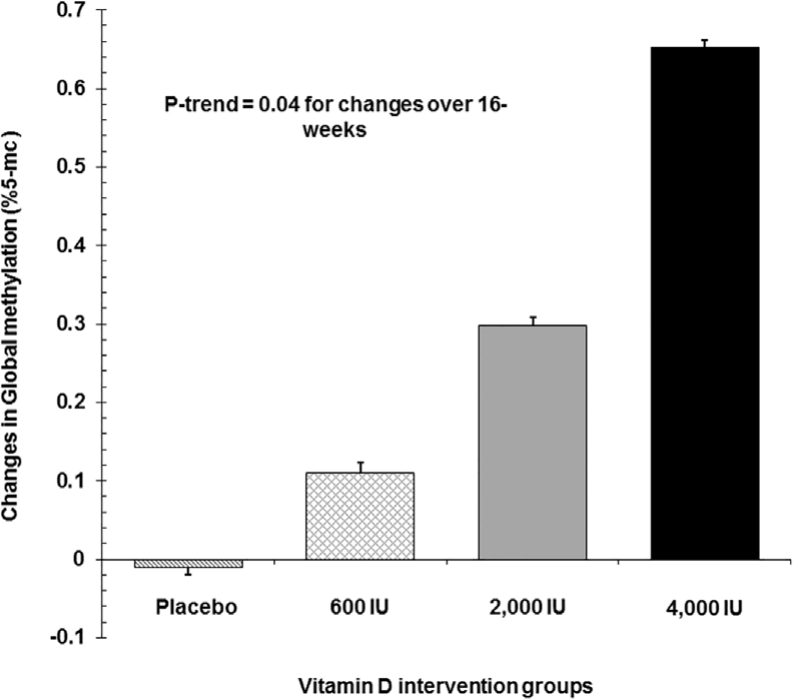
Changes in global methylation in response to 16 week vitamin D3 supplementation (placebo, 600 IU/d, 2,000 IU/d, or 4,000 IU/d). Data represent mean ± SE. *p* trend <0.01.

As seen in the cross-sectional LACHY study, at the baseline, circulating 25(OH)D level was also positively correlated with level of global %5-mC (r = 0.31, *p* = 0.02), that is, lower 25(OH)D was associated with global hypomethylation. As we previously reported, a significant increase was observed in the changes of serum 25(OH)D from baseline to 16 weeks (0.9 ± 2.0, placebo; 8.6 ± 2.1, 600 IU/day; 20.1 ± 2.0, 2,000 IU/day; and 21.2 ± 2.0 ng/ml, 4,000 IU/day; *p*<0.01).[13] In parallel, the 16 week of vitamin D_3_ supplementation increased global %5-mC in a dose-dependent manner (-0.01 ± 0.01%, placebo; 0.11 ± 0.01%, ~600 IU/day; 0.30 ± 0.01%, ~2,000 IU/day; and 0.65 ± 0.01%, ~4,000 IU/day group; *p*-trend = 0.04, Fig 1). Furthermore, changes in global %5-mC were significantly correlated with changes in 25(OH)D (r = 0.60, *p* = 0.02, S1 Fig).

There was no correlation between the composition of leukocytes (three major cell types: neutrophils, lymphocytes and monocytes) and 25OHD level or %5-mC at the baseline (all *Ps* > 0.05, Table A in S1 File). In addition, no correlation was found between change in composition of leukocytes and change in 25OHD or change in %5-mC (all *Ps* > 0.05, Table B in S1 File). Therefore, no leukocyte composition variables as potential covariates were included in the model.

## Discussion

To the best of our knowledge, this is the first study to examine the relationship between vitamin D and global DNA methylation using a two-stage design (cross-sectional observation followed by RCT). Our major findings include: 1) African Americans generally have lower level of global methylation than Caucasians; 2) low 25OHD levels are associated with global hypomethylation in African American individuals; 3) vitamin D3 supplementation increases levels of global DNA methylation in a dose-dependent manner in young African Americans with vitamin D deficiency.

To date, only a few human studies have been conducted to examine the relationship between vitamin D level and global methylation, and mainly in Caucasian populations. Nair-Shalliker *et al*.[5] reported that exposure to solar UV radiation was inversely associated with global DNA methylation and this association was not influenced by vitamin D level in healthy adults in South Australia. Chavez Valencia *et al*.[6] exposed primary human blood mononuclear cells to vitamin D (calcitriol) for up to 120 hours, and measured genome-scale DNA methylation response using the Illumina Infinium HumanMethylation450 beadchip and reported no significant DNA methylation changes in response to vitamin D treatment. In a clinical trial conducted by Hubner *et al*,[7] 50 German subjects (median age 68.0 years) were supplemented with a daily oral dose of B vitamins (500 ug folic acid, 500 ug vitamin B12 and 50 mg vitamin B6), 1200IU vitamin D and 456 mg calcium for one year. Long interspersed nucleotide element-1 (LINE-1) methylation was determined in genomic DNA from blood cells as a surrogate for whole genome methylation. The combined vitamin B and D supplementation had no effect on LINE-1 methylation, especially in subjects with no severe vitamin deficiency.[7] Tapp *et al*. investigated the effects of nutritional factors on age-related DNA methylation in the human rectal mucosa samples and reported a weak positive correlation between vitamin D and LINE-1 methylation.[8] In the present study, we showed that low 25(OH)D is associated with global hypomethylation only in African Americans, not in Caucasians. Moreover, vitamin D3 supplementation increases levels of global DNA methylation in a dose-dependent manner in young African Americans with vitamin D deficiency. The new findings provide further support for our previous observations that vitamin D deficiency is associated with leukocyte DNA methylation changes. Subjects with vitamin D deficiency tend to have reduced levels of DNA methylation of the top 10 differentially methylated CpG sites compared with controls.[16]

Leukocytes consist of a mixture of different cell types that all may have a cell-specific epigenome. However, Talens *et al*.[17] demonstrated that for the large majority of candidate loci, inter-individual differences in the cellular composition of the blood sample did not contribute to the variation observed in DNA methylation or explained only a minor proportion of that variation. In the present study, we did not find correlation between the change in composition of leukocytes (i.e. neutrophils, lymphocytes and monocytes) and either change in 25(OH)D or change in global DNA methylation suggesting that increased global methylation induced by vitamin D3 supplementation might not be biased by the different composition of leukocytes.

Racial influence on the methylome has been reported previously. African Americans are generally at hypomethylated state compared to Caucasians.[9–11] Our cross-sectional LACHY study confirms the previous findings. In the present study, we observed a significant interaction between race and 25(OH)D on global methylation. However this is not unexpected since over 97% of the Caucasians were vitamin D deficient, whereas 66% of African Americans were vitamin D deficient. Whether the difference in global methylation between Caucasians and African Americans were race dependent or vitamin D dependent warrants further investigation. African Americans suffer from disproportionately high rates of cardiovascular disease and cancer. Altered methylome may be one of underlying mechanisms that explain these health disparities.

Global genomic hypomethylation is a common epigenetic event in cancer that mostly results from hypomethylation of repetitive DNA elements. Loss of global methylation may lead to chromosomal instability, loss of imprinting, and activation of transposable elements, which lead to disturbances in the genome.[1] The mechanism underlying the effect of vitamin D on global methylation is not well understood. Recent evidence suggests that vitamin D can induce DNA demethylation in the promoter of certain tumor suppressor genes,[8,18] while increasing global DNA methylation of LINE-1.[8] Interestingly, the interaction of vitamin D and DNA methylation is mediated by the expression of GADD45 (growth arrest and DNA damage).[19–21] GADD45A is one of the enzymes that relieves epigenetic gene silencing by promoting DNA repair and promotes global DNA demethylation.[22] Further research is needed to better understand how vitamin D regulates the epigenome.

Major strengths in the present study included: 1) Our large cross-sectional cohort with racial diversity allows us to determine the race/ethnic differences in vitamin D and global methylation relation; 2) The dose-responsive RCT of vitamin D supplementation not only allowed us to validate our findings obtained from the cross-sectional study, but also helped to establish causal and dose-response relationships; 3) Only inclusion of African American participants with vitamin D deficiency in the dose-responsive RCT of vitamin D supplementation maximized the chance to detect the biological effect of vitamin D. We acknowledge there are also limitations in the study. First, the sample size in each group of the RCT of vitamin D supplementation was relatively small. Therefore, large RCTs of vitamin D supplementation are needed to validate our findings. Second, we have only analyzed the effect of three major leukocyte cell types (neutrophils, lymphocytes and monocytes) on the vitamin D-DNA methylation relation, the influence of other subtypes of leukocytes remains unknown and warrants further investigation.

## Conclusion

African Americans have lower level of global DNA methylation compared to Caucasians. In addition, low vitamin D level is associated with global hypomethylation in African American individuals, whereas vitamin D supplementation increases global methylation in a dose-responsive manner. The present study provides an important piece of evidence that vitamin D plays a role in epigenetic regulations in humans. Moreover, our study suggests that vitamin D may exert its impact on health disparity through epigenomic regulation.

## Supporting Information

S1 Fig Correlation between changes in 5-methylcytosine and 25(OH)D in the 4,000 IU group. (TIF)

S1 File Table A. Baseline associations of serum 25(OH)D and 5-methylcytosine with composition of leukocytes in all participants. Table B. Associations of changes in serum 25(OH)D and 5-methylcytosine with changes in composition of leukocytes in all participants. Table C. Raw 5-methylcytisine data in the vitamin D3 supplementation trial.
(DOCX)
