# Correction: Race/Ethnicity-Specific Association of Vitamin D and Global DNA Methylation: Cross-Sectional and Interventional Findings

**DOI:** 10.1371/journal.pone.0162582

**Published:** 2016-09-01

**Authors:** Haidong Zhu, Jigar Bhagatwala, Ying Huang, Norman K. Pollock, Samip Parikh, Anas Raed, Bernard Gutin, Gregory A. Harshfield, Yanbin Dong

There is an error in the fifth sentence of the fourth paragraph of the Discussion. The correct sentence is: However this is not unexpected since over 97% of the Caucasians were vitamin D sufficient, whereas 66% of African Americans were vitamin D deficient.

There is an error in the caption for Fig 1, "Changes in global methylation in response to 16 week vitamin D3 supplementation (placebo, 600 IU/d, 2,000 IU/d, or 4,000 IU/d)." Please see the complete, correct Fig 1 caption here.

**Fig 1:**
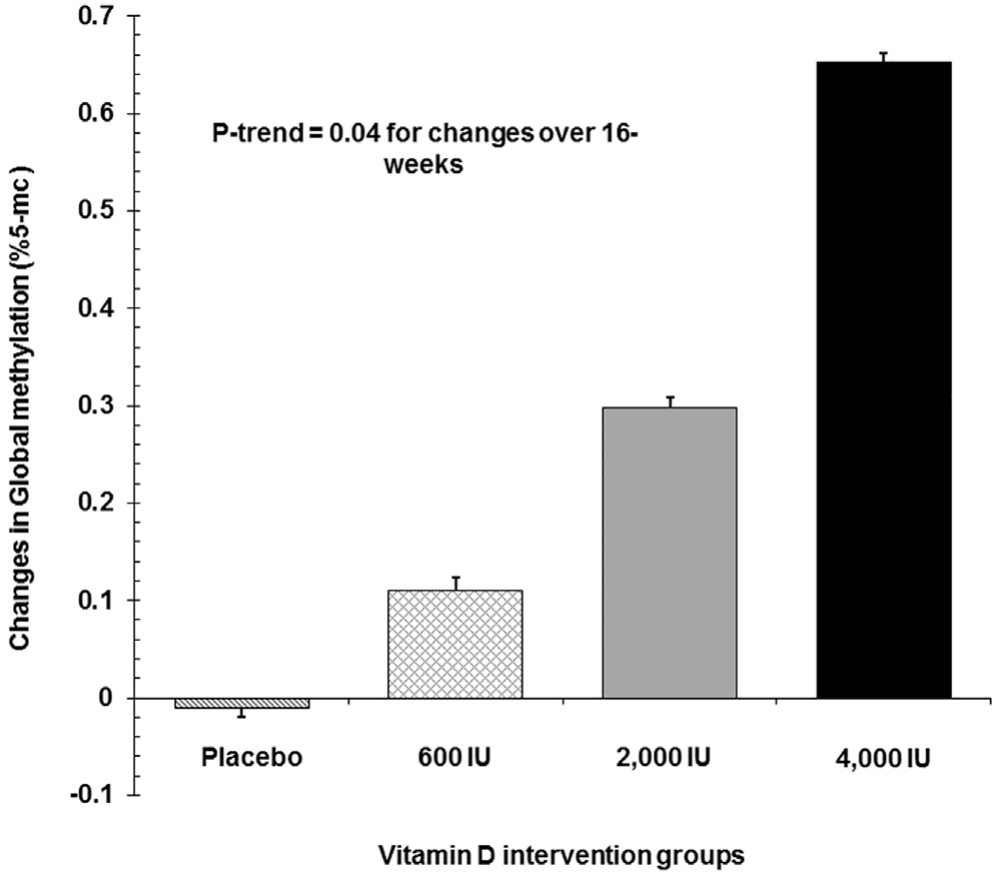
Changes in global methylation in response to 16 week vitamin D3 supplementation (placebo, 600 IU/d, 2,000 IU/d, or 4,000 IU/d). Data represent mean ± SE. p trend <0.04.
