# Supplementary figures and images for: Race/Ethnicity-Specific Association of Vitamin D and Global DNA Methylation: Cross-Sectional and Interventional Findings
Source: PLoS One. 2016 Apr 6;11(4):e0152849. doi: 10.1371/journal.pone.0152849 (PMC4822838; doi:10.1371/journal.pone.0152849)

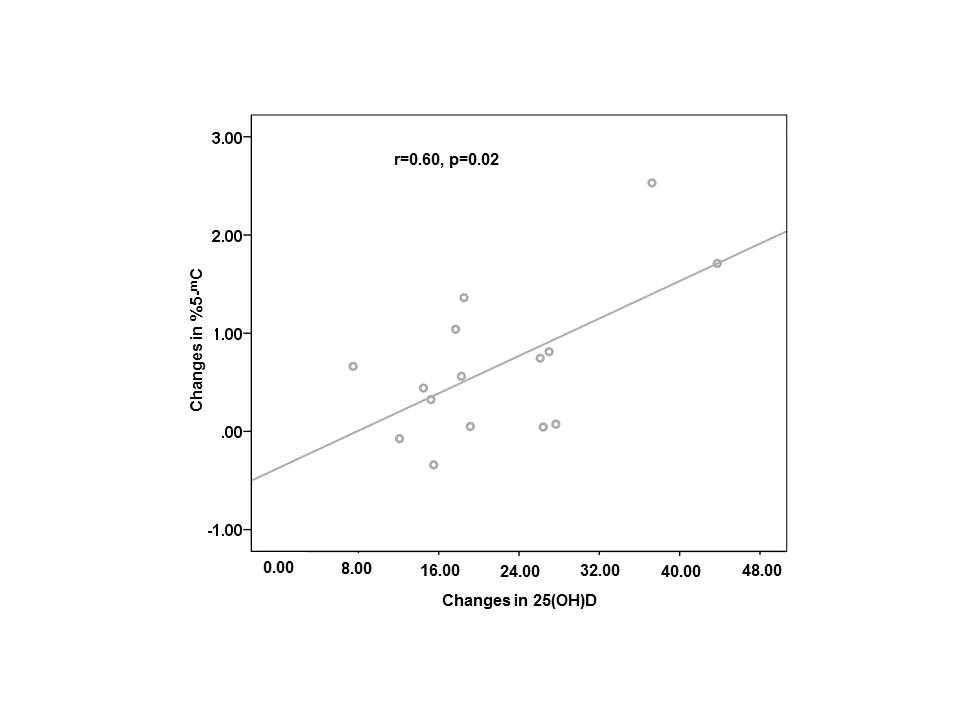

Supplement: S1 Fig — (TIF) [file pone.0152849.s001.tif]
