# Supplementary material for: Race/Ethnicity-Specific Association of Vitamin D and Global DNA Methylation: Cross-Sectional and Interventional Findings
Source: PLoS One. 2016 Apr 6;11(4):e0152849. doi: 10.1371/journal.pone.0152849 (PMC4822838; doi:10.1371/journal.pone.0152849)
Supplement: S1 File — Table A. Baseline associations of serum 25(OH)D and 5-methylcytosine with composition of leukocytes in all participants. Table B. Associations of changes in serum 25(OH)D and 5-methylcytosine with changes in composition of leukocytes in all participants. Table C. Raw 5-methylcytisine data in the vitamin D3 supplementation trial. (DOCX) [file pone.0152849.s002.docx]

**Table A.** Baseline associations of serum 25(OH)D and 5-methylcytosine with composition of leukocytes in all participants^*^

| Baseline %5-mC  Baseline 25(OH)D |  |  |  |  |  |
| --- | --- | --- | --- | --- | --- |
|  | *r* | *P*-value | *r* | *P*-value |  |
| % Neutrophils | -0.04 | 0.79 | -0.07 | 0.60 |  |
| % Lymphocytes | 0.09 | 0.51 | -0.01 | 0.99 |  |
| % Monocytes | -0.19 | 0.16 | -0.11 | 0.44 |  |

Abbreviations: 25(OH)D, 25-hydroxyvitamin D; 5-mC, 5-methylcytosine.

*^*^*Pearson’s bivariate correlations were used to examine relationships of the changes in serum 25(OH)D and %5-mC levels with changes in the composition of leukocytes.

**Table B.** Associations of changes in serum 25(OH)D and 5-methylcytosine with changes in composition of leukocytes in all participants^*^

| Change in %5-mC  Change in 25(OH)D |  |  |  |  |  |
| --- | --- | --- | --- | --- | --- |
|  | *r* | *P*-value | *r* | *P*-value |  |
| Change in % Neutrophils | -0.05 | 0.76 | 0.23 | 0.13 |  |
| Change in % Lymphocytes | 0.03 | 0.82 | -0.25 | 0.09 |  |
| Change in % Monocytes | -0.15 | 0.31 | 0.05 | 0.76 |  |

Abbreviations: 25(OH)D, 25-hydroxyvitamin D; 5-mC, 5-methylcytosine.

*^*^*Pearson’s bivariate correlations were used to examine relationships of the changes in serum 25(OH)D and %5-mC levels with changes in the composition of leukocytes.

**Table C**. Raw 5-methylcytosine data in the vitamin D3 supplementation trial

|  | ID | sex, m=1, f=2 | Age | 5-mC pre | 5-mC post |
| --- | --- | --- | --- | --- | --- |
|  | 1 | 2 | 21.35 | 1.056 | 3.949 |
|  | 2 | 1 | 25.39 | 0.025 | 2.556 |
|  | 3 | 1 | 20.74 | 0.418 | 1.281 |
|  | 4 | 2 | 44.42 | 1.281 | 1.913 |
|  | 5 | 2 | 32.82 | 2.321 | 0.231 |
|  | 6 | 2 | 33.77 | 0.745 | 0.209 |
|  | 7 | 2 | 28.42 | 0.062 | 0.402 |
|  | 8 | 1 | 17.93 | 2.192 | 1.602 |
|  | 9 | 2 | 13.17 | 0.338 | 0.411 |
|  | 10 | 2 | 35.65 | 0.193 | 0.504 |
|  | 11 | 2 | 29.56 | 2.288 | 0.389 |
|  | 12 | 2 | 15.94 | 0.863 | 0.756 |
|  | 13 | 2 | 28.35 | 1.441 | 1.442 |
|  | 14 | 2 | 27.01 | 0.278 | 1.988 |
|  | 15 | 2 | 32.88 | 1.581 | 2.128 |
|  | 16 | 1 | 25.59 | 1.122 | 1.174 |
|  | 17 | 1 | 28.21 | 0.617 | 1.217 |
|  | 18 | 2 | 20.01 | 0.025 | 3.714 |
|  | 19 | 2 | 17.61 | 1.502 | 2.386 |
|  | 20 | 2 | 36.62 | 2.212 | 0.817 |
|  | 21 | 2 | 35.69 | 0.109 | 0.418 |
|  | 22 | 1 | 14.72 | 0.672 | 1.066 |
|  | 23 | 2 | 42.18 | 0.615 | 0.644 |
|  | 24 | 1 | 13.98 | 0.07 | 0.07 |
|  | 25 | 2 | 45.83 | 0.344 | 0.344 |
|  | 26 | 2 | 17.84 | 0.394 | 0.052 |
|  | 27 | 2 | 16.47 | 0.025 | 0.248 |
|  | 28 | 2 | 25.85 | 0.543 | 0.867 |
|  | 29 | 2 | 15.6 | 0.618 | 0.555 |
|  | 30 | 2 | 41.39 | 0.244 | 0.587 |
|  | 31 | 1 | 20.63 | 0.917 | 0.966 |
|  | 32 | 2 | 15.05 | 0.468 | 0.369 |
|  | 33 | 2 | 19.23 | 0.618 | 1.178 |
|  | 34 | 1 | 17.95 | 0.169 | 0.468 |
|  | 35 | 2 | 19.78 | 0.792 | 0.362 |
|  | 36 | 2 | 40.3 | 0.269 | 0.817 |
|  | 37 | 2 | 22.43 | 0.547 | 0.554 |
|  | 38 | 1 | 25.12 | 0.025 | 0.274 |
|  | 39 | 2 | 19.83 | 0.025 | 0.249 |
|  | 40 | 2 | 30.17 | 0.539 | 0.672 |
|  | 41 | 2 | 20.72 | 0.025 | 0.77 |
|  | 42 | 1 | 17 | 0.025 | 0.025 |
|  | 43 | 2 | 16.81 | 0.025 | 0.025 |
|  | 44 | 2 | 33.52 | 0.038 | 0.267 |
|  | 45 | 2 | 32.05 | 0.333 | 0.025 |
|  | 46 | 2 | 32.35 | 0.186 | 0.525 |
|  | 47 | 2 | 20.39 | 0.053 | 0.097 |
|  | 48 | 2 | 43.28 | 0.37 | 0.171 |
|  | 49 | 2 | 35.89 | 0.77 | 1.432 |
|  | 50 | 2 | 25.06 | 0.127 | 0.362 |
|  | 51 | 2 | 20.35 | 0.393 | 0.834 |
|  | 52 | 2 | 16.02 | 0.458 | 0.65 |
|  | 53 | 2 | 30.64 | 0.045 | 0.79 |
|  | 54 | 2 | 41.16 | 0.173 | 0.753 |
|  | 55 | 2 | 41.35 | 0.18 | 0.106 |
|  | 56 | 2 | 19.42 | 0.075 | 1.153 |
|  | 57 | 2 | 17.54 | 0.474 | 1.286 |
|  | 58 | 2 | 13.44 | 0.05 | 0.467 |
